# Silver Nanorods Wrapped with Ultrathin Al_2_O_3_ Layers Exhibiting Excellent SERS Sensitivity and Outstanding SERS Stability

**DOI:** 10.1038/srep12890

**Published:** 2015-08-12

**Authors:** Lingwei Ma, Yu Huang, Mengjing Hou, Zheng Xie, Zhengjun Zhang

**Affiliations:** 1 State Key Laboratory of New Ceramics and Fine Processing, School of Materials Science and Engineering, Tsinghua University, Beijing 100084, P.R. China; 2 Key Laboratory of Advanced Materials (MOE), School of Materials Science and Engineering, Tsinghua University, Beijing 100084, P.R. China; 3 High-Tech Institute of Xi’an, Shannxi 710025, P.R. China

## Abstract

Silver nanostructures have been considered as promising substrates for surface-enhanced Raman scattering (SERS) with extremely high sensitivity. The applications, however, are hindered by the facts that their morphology can be easily destroyed due to the low melting points (~100 °C) and their surfaces are readily oxidized/sulfured in air, thus losing the SERS activity. It was found that wrapping Ag nanorods with an ultrathin (~1.5 nm) but dense and amorphous Al_2_O_3_ layer by low-temperature atomic layer deposition (ALD) could make the nanorods robust in morphology up to 400 °C, and passivate completely their surfaces to stabilize the SERS activity in air, without decreasing much the SERS sensitivity. This simple strategy holds great potentials to generate highly robust and stable SERS substrates for real applications.

Surface-enhanced Raman scattering (SERS) has emerged into an important analytical technique in biological, clinical, chemical and environmental sensing for its superior capability to nondestructively provide plenteous structural information of analytes at extremely low amounts^1^,^2^,^3^,^4^,^5^,^6^,^7^. The reason is that the Raman scattering cross-section of molecules can be enhanced by many orders of magnitude when they are adsorbed on the very surface of noble metals (e.g., Au, Ag and Cu) with nanometer scale roughness, aroused from electromagnetic (EM) enhancement and chemical (CM) enhancement^8^,^9^. In general, the SERS effect is a combination of both and depends significantly on the morphology of nanostructures^10^,^11^. Tremendous efforts have thus been devoted in the past years to studying the relationship between the SERS effect and the morphological features of noble metals, and to fabricating novel nanostructures to boost real SERS applications^12^,^13^,^14^.

Ag nanostructures especially one-dimensional (1D) structures such as nanorods, nanowires, etc., have long been considered as the most promising candidate for SERS substrates due to their high plasmonic efficiency and EM enhancement effect, as well as the lower cost versus Au^15^,^16^,^17^. However, they suffer from a low melting point (~100 °C), which drops a lot for materials with reduced dimensions in comparison with that of their bulk counterparts^18^,^19^,^20^,^21^,^22^. This causes the morphological instability of Ag nanostructures and as a result, may deteriorate their SERS performance during Raman sensing, especially at high-temperature conditions^23^,^24^,^25^,^26^,^27^. Ag nanostructures suffer also from the highly active surfaces that are readily oxidized/sulfured in air, leading to a rapid decay in SERS sensitivity^28^,^29^,^30^. Therefore, a prerequisite for successful applications of Ag nanostructures in practical SERS-based sensors is to devise methodologies to make their morphology robust at high temperatures and to passivate their surfaces under atmospheric conditions.

It has been reported that coating Ag nanostructures with protective metal oxide layers was able to suppress their surface reactions with air^23^,^25^,^28^,^29^, and might be helpful to preserve their morphology at elevated temperatures^31^,^32^. For example, coating Ag islands with Al_2_O_3_ films could maintain their SERS activity for a period of 34 days^29^; coating Ag nanorods with TiO_2_ thin films by glancing angle deposition (GLAD) technique could sustain their morphology and stabilize the SERS performance at 100 °C (above which coalescence of Ag nanorods was observed)^31^. However, a drawback of this coating approach is the giant decrease in SERS sensitivity, which is caused by the coating layers that separate the target molecules from Ag nanostructures^29^,^33^,^34^, and by the possible morphology changes of silver created during coating process. It is therefore highly demanded to find ways to deposit protective layers that completely cover Ag nanostructures at relatively low temperatures to maintain their morphological features, with a precise control of the coating thickness to prohibit sharply decreasing the SERS sensitivity but meanwhile thick enough to make them robust against temperature.

In this study, we employed atomic layer deposition (ALD) technique to prepare ultrathin Al_2_O_3_ films that fully wrapping Ag nanorods at a temperature of 50 °C, and investigated the coating influences on the morphological stability of Ag nanorods at elevated temperatures in air, as well as their SERS sensitivity and activity at ambient conditions. It was found that an ultrathin (~1.5 nm) but dense and amorphous Al_2_O_3_ layer was thick enough to make Ag nanorods robust in morphology to a temperature of 400 °C, and passivate sufficiently their surfaces to stabilize the SERS activity in air, without largely decreasing the SERS sensitivity.

## Results and Discussion

**Figure 1:**
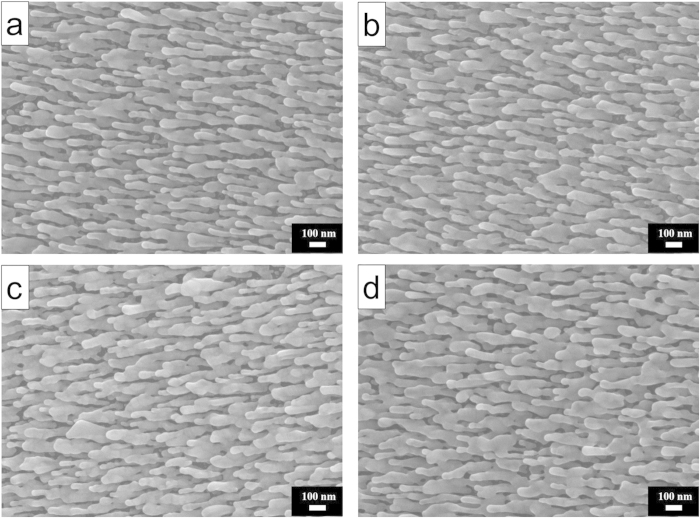
SEM images of (**a**) as-prepared Ag nanorods; and those heated in the ALD chamber at (**b**) 50 °C, (**c**) 80 °C and (**d**) 100 °C, respectively.

Figure 1a shows a typical SEM micrograph of the as-deposited Ag nanorods, from which one sees clearly that these slanted nanorods are well separated. In order to find an appropriate deposition temperature at which morphology changes of Ag nanorods can be avoided during ALD processing, we heated the as-deposited Ag nanorods in the ALD chamber at temperatues of 50, 80 and 100 °C for 8 minutes, separately, without purging the ALD precursors. Figure 1b–d show the SEM images of Ag nanorods after thermal treatment at the three temperatures. In comparison with the as-deposited nanorods, no noticeable morphology change was observed for those heated at 50 °C. For the samples annealed at 80 and 100 °C, however, partial melting of Ag nanorods was observed, which resulted in adhesion of neighboring nanorods. Since the SERS effect is very morphology-dependent, this may cause sharp differences in their SERS sensitivity^35^,^36^. Figure S1 compares the average diameters and SERS performance of the above four samples. It is seen that Ag nanorods became larger after heated at 80 and 100 °C and declined in SERS sensitivity, using 5 × 10^−6^ M methylene blue (MB) as the probe molecule, while the nanorods heated at 50 °C were of a similar SERS sensitivity as the as-deposited ones. We thus selected 50 °C to prepare Al_2_O_3_ coatings by ALD approach.

**Figure 2:**
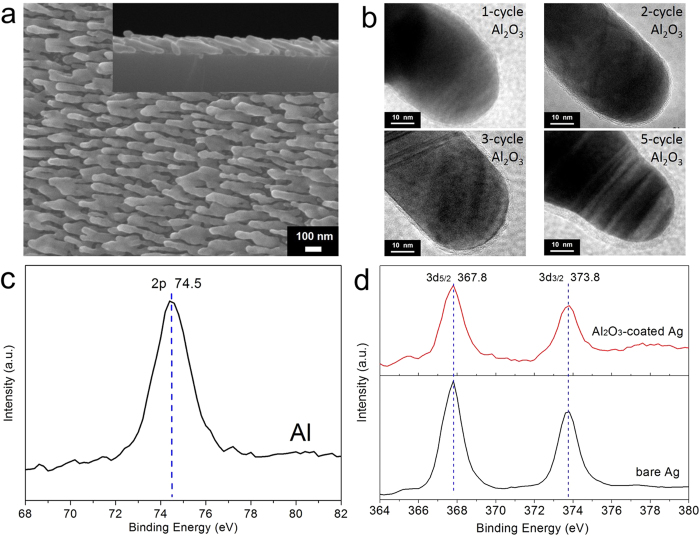
(**a**) A typical top-view SEM image of Ag nanorods coated with Al_2_O_3_ by 5 ALD cycles at 50 °C; inset is a corresponding side-view SEM image. (**b**) HRTEM images of the Ag nanorods coated with Al_2_O_3_ layers by 1, 2, 3 and 5 ALD cycles. (**c**) A typical Al 2p XPS spectrum of the Al_2_O_3_ layer by 5 ALD cycles. (**d**) Ag 3d_5/2_ and Ag 3d_3/2_ XPS spectra of uncoated Ag nanorods and coated ones by 5 ALD cycles.

Al_2_O_3_ thin films of various thickness were deposited on Ag nanorods at 50 °C by changing the ALD cycle numbers to 1, 2, 3 and 5, respectively. Figure 2a shows a typical top-view SEM image of the Ag nanorods after coating by 5 ALD cycles; inset is a corresponding side-view SEM image. One sees that the nanorods are ~30 nm in diameter, ~280 nm in length, and are well-separated, without apparent variation in morphology compared with the as-deposited ones (see Fig. 1a). Figure 2b shows the HRTEM images of individual Ag nanorods coated with Al_2_O_3_ layers by 1, 2, 3 and 5 ALD cycles. It is seen that the coating layers are amorphous in structure and of different thickness (from <1 nm to ~4 nm), fully wrapping the nanorods. A linear relationship between the thickness of Al_2_O_3_ layers and ALD cycle numbers was observed and plotted in Figure S2, from which a deposition rate of ~0.7 nm/cycle was derived. This suggests a precise control of the layer thickness to sub-nanometer scale by this approach^33^,^37^,^38^. The chemical states of the Ag nanorods and Al_2_O_3_ shells were analyzed by XPS. Figure 2c shows a typical Al 2p XPS spectrum (calibrated with reference to the C1s peak at 284.8 eV) of the coating layer deposited by 5 ALD cycles. The peak is located at ~74.5 eV, indicating the formation of Al-O bond in Al_2_O_3_^39^,^40^. In addition, the Ag 3d_5/2_ and Ag 3d_3/2_ doublet peaks of this coated sample and the uncoated one are both centered at ~367.8 and ~373.8 eV, respectively, see Fig. 2d. This is good in agreement with those of elemental Ag^41^,^42^, and indicates that there was no destruction of Ag by ALD processing^30^,^37^ and that the coating layer by 5 cycles was very thin.

**Figure 3:**
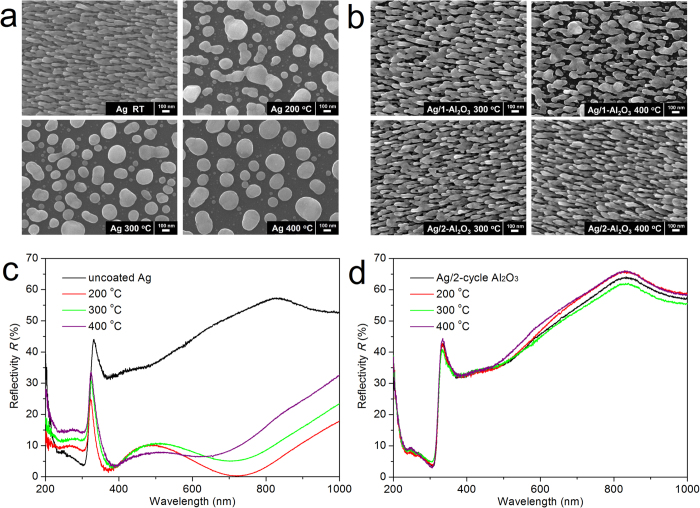
SEM images of (**a**) uncoated Ag nanorods before/after annealing at 200, 300 and 400 °C; and (**b**) Ag nanorods coated with Al_2_O_3_ layers by 1 and 2 ALD cycles before/after annealing at 300 and 400 °C, respectively. Reflectance spectra of (**c**) uncoated Ag nanorods; and (**d**) Ag nanorods coated with an Al_2_O_3_ layer by 2 ALD cycles before/after annealing at 200, 300 and 400 °C, respectively.

The influence of Al_2_O_3_ layers on the morphological stability of Ag nanorods was investigated by thermally annealing these samples in air at temperatures of 200, 300 and 400 °C for 30 minutes. It was found that the uncoated Ag nanorods melted completely at these temperatures, evolving from irregular aggregations to spherical particles as the annealing temperature rising, see Fig. 3a. Figure 3b shows the SEM images of Ag nanorods coated by 1 and 2 ALD cycles, after annealing at 300 and 400 °C, separately. The nanorods coated by 1 ALD cycle (the Al_2_O_3_ layer was <1 nm thick) were robust in morphology at 200 °C (not shown), but melted partly at 300 and 400 °C. For the nanorods coated by 2 ALD cycles (the Al_2_O_3_ layer was ~1.5 nm thick), however, no obvious morphological change was observed after being heated at 300 and 400 °C, in comparison with the as-deposited ones. Similar results were also obtained for Ag nanorods coated by 3 and 5 ALD cycles, see Figure S3. From literature, it is known that any morphological variation of Ag nanorods may cause sharp differences in their optical reflectance and SERS sensitivity^43^,^44^,^45^. Figure 3c,d and S4 show respectively the reflectance spectra of uncoated Ag nanorods and coated ones by 1, 2, 3 and 5 ALD cycles, before/after thermal treatment. Due to the large morphological changes of the uncoated sample after annealing (Fig. 3a), their reflectance spectra were very different from those before annealing. For the nanorods coated by 2 or more ALD cycles, their reflectance spectra remained almost unchanged after annealing, which is in good agreement with their robust morphology shown by Fig. 3b and S3. As for the nanorods coated by only 1 ALD cycle, the reflectance spectrum remained unchanged at 200 °C, and varied slightly but obviously at 300 and 400 °C. This is due to their morphology changes shown by Fig. 3b and indicates that an Al_2_O_3_ layer of <1 nm was too thin to protect the morphology of Ag nanorods at temperatures >200 °C.

**Figure 4:**
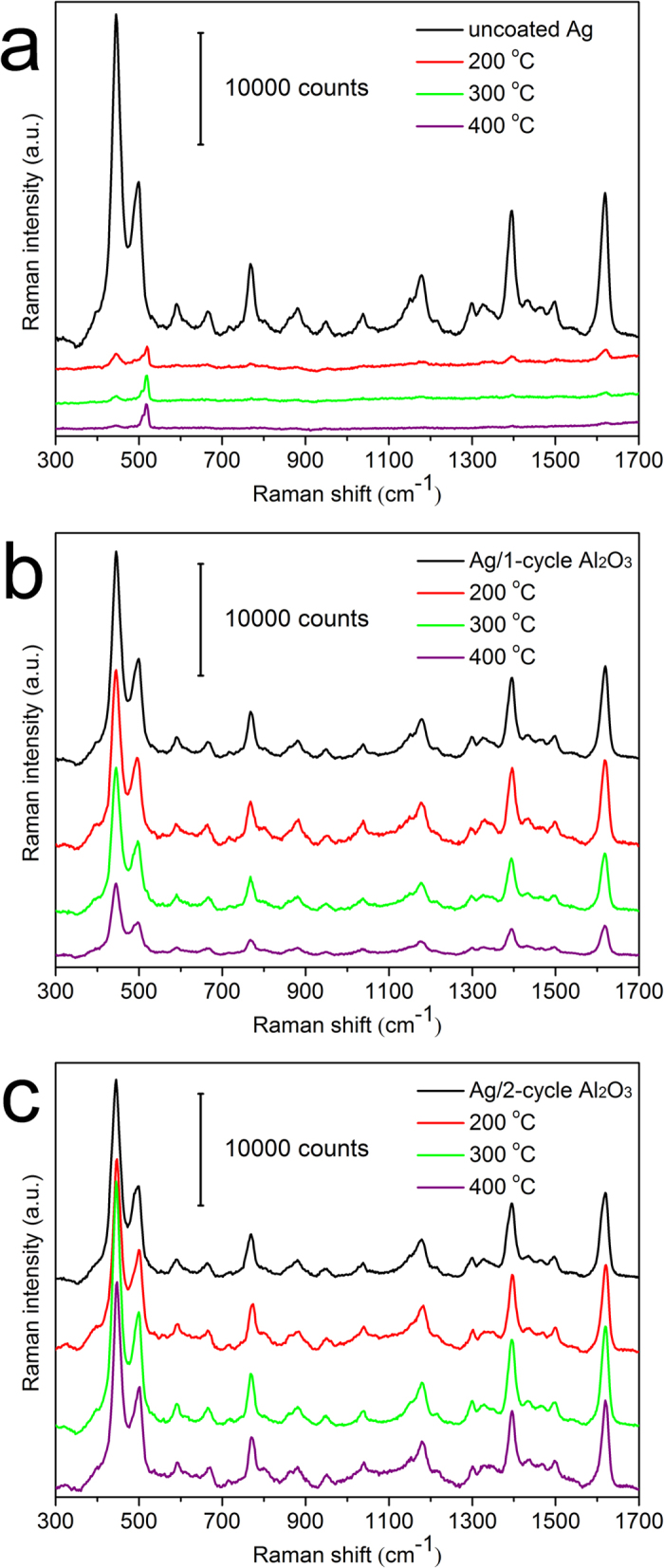
Raman spectra of 5 × 10^−6^ M MB adsorbed on (**a**) uncoated Ag nanorods; and on Ag nanorods coated with Al_2_O_3_ layers by (**b**) 1 ALD cycle and (**c**) 2 ALD cycles, before/after annealing at 200, 300 and 400 °C, respectively.

The SERS performance of the above Ag nanorods before/after annealing was evaluated using 5 × 10^−6^ M MB as the probing molecule. Figure 4a compares the Raman spectra of MB on uncoated Ag nanorods as SERS substrate, before/after annealing at 200, 300 and 400 °C, respectively. It is seen that the Raman intensity of MB on annealed, uncoated Ag nanorods dropped drastically, owing to the deterioration of their morphology, see Fig. 3a. For nanorods coated by 1 ALD cycle, see Fig. 4b, their SERS sensitivity remained almost unchanged after annealing at 200 °C, and decreased somehow at 300 and 400 °C because of the slight change of their morphology (see Fig. 3b). For the Ag nanorods coated by 2 ALD cycles, see Fig. 4c, their SERS sensitivity remained almost constant after annealing at 200, 300 and 400 °C. These results suggest that an ultrathin (~1.5 nm) Al_2_O_3_ layer by 2 ALD cycles was effective to protect Ag nanorods in both morphology stiffness and SERS sensitivity up to a temperature of 400 °C in air.

**Figure 5:**
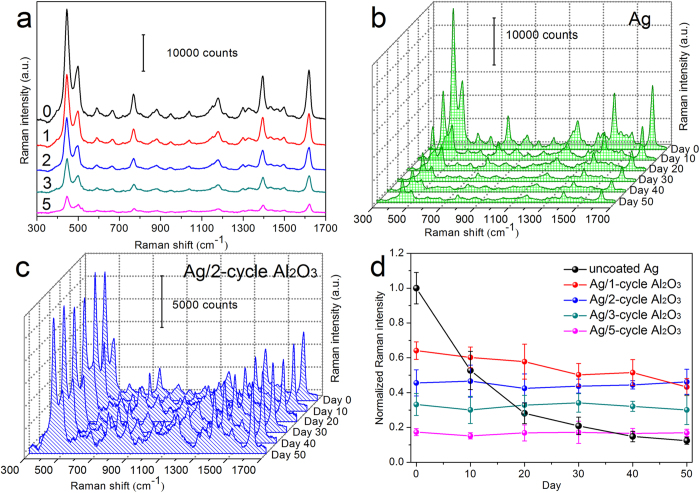
(**a**) Raman spectra of 5 × 10^−6^ M MB adsorbed on uncoated Ag nanorods (0 cycle) and on Ag nanorods coated with Al_2_O_3_ layers by 1, 2, 3 and 5 ALD cycles. Raman spectra of 5 × 10^−6^ M MB adsorbed on (**b**) uncoated Ag nanorods and (**c**) on Ag nanorods coated with an Al_2_O_3_ layer by 2 ALD cycles, measured in 50 days. (**d**) The normalized Raman intensities of MB Raman peak at 1622 cm^−1^ as a function of the measurement time.

The effect of Al_2_O_3_ coating layers on the SERS activity of Ag nanorods at room temperature in air was also investigated as a function of time. As the starting point, the Raman spectra of 5 × 10^−6^ M MB on as-prepared, uncoated Ag nanorods, and on as-prepared, coated Ag nanorods by 1, 2, 3 and 5 ALD cycles were measured and compared as references, see Fig. 5a. As expected, all coated nanorods exhibited strong SERS sensitivity, and a gradual decrease in the Raman signal intensity of MB was observed with an increase in the thickness of Al_2_O_3_ coating layers^32^. Afterwards, the Raman spectra of MB were measured again in every 10 days using the above samples as SERS substrates (stored in air). Figure 5b,c show Raman spectra of MB on uncoated nanorods and on nanorods coated by 2 ALD cycles, respectively, measured at different times. One sees that the SERS sensitivity of the uncoated substrate dropped rapidly in air, while that of the substrate coated by Al_2_O_3_ by 2 ALD cycles remained almost constant. For a clear comparison, we normalized the intensity of the peak of MB at 1622 cm^−1^ obtained on various SERS substrates to that obtained on the as-prepared, uncoated Ag nanorods, and plotted in Fig. 5d as a function of time. It shows that the SERS sensitivity of uncoated Ag nanorods dropped drastically in air, and was about one order smaller after 50 days, while that of nanorods coated by 2 and more ALD cycles remained at the same level. This indicates that the stability of the SERS sensitivity of Ag nanorods in air was greatly improved by Al_2_O_3_ coating. It is also noticed that after coating with an Al_2_O_3_ layer by 2 ALD cycles (~1.5 nm thick), the SERS sensitivity of Ag nanorods was still ~50% that of the as-prepared, uncoated ones. For nanorods coated with a thinner layer (<1 nm thick) by only 1 ALD cycle, their SERS sensitivity was a little higher at the beginning, but decreased ~18% after 50 days in air, i.e., their SERS stability was not as good as that of Ag nanorods coated by 2 ALD cycles or more. These suggest that a ~1.5 nm Al_2_O_3_ layer be optimal to combine both excellent SERS sensitivity and outstanding SERS stability of Ag nanorods in air.

**Figure 6:**
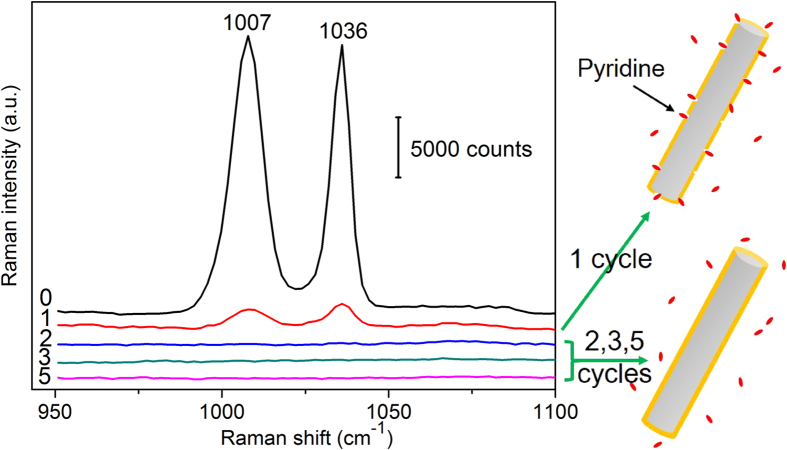
Raman spectra of 1 × 10^−2^ M pyridine adsorbed on uncoated Ag nanorods (0 cycle) and on Ag nanorods coated with Al_2_O_3_ layers by 1, 2, 3 and 5 ALD cycles, respectively. The schematic shows that pinholes were detected in the Al_2_O_3_ layer by 1 ALD cycle, but not in those by 2 or more ALD cycles.

The reason why an Al_2_O_3_ coating layer deposited by 1 ALD cycle (<1 nm thick) was not capable to effectively protect Ag nanorods is intriguing. It is known that pyridine has a small size and a relatively large Raman cross-section and can be adsorbed on the surface of Ag nanostructures intensively. Therefore, it can be used as a mark to check out whether the Al_2_O_3_ coating layers were compact enough to protect Ag nanorods. If there are any pinholes in the Al_2_O_3_ layer, pyridine could penetrate through pinholes and be adsorbed on the surface of Ag nanorods, thus exhibiting Raman siganls^46^,^47^,^48^. We dipped the as-prepared Ag nanorods (uncoated, and coated by 1, 2, 3 and 5 ALD cycles) into a 1 × 10^−2^ M pyridine solution, and measured the Raman spectra. Figure 6 shows Raman spectra of pyridine on different substrates. It is seen that Raman spectra of pyridine showed up only on uncoated Ag nanorods and on nanorods coated by 1 ALD cycle. On those coated by 2 and more ALD cycles, no Raman signal of pyridine was observed. These suggest that an Al_2_O_3_ layer of <1 nm thick (by 1 ALD cycle deposition) was too thin to avoid pinholes, and was not dense enough to protect effectively Ag nanorods, and confirm again that an Al_2_O_3_ layer of ~1.5 nm thick was an optimal choice by this approach.

In conclusion, we developed a low-temperature ALD approach to completely cover Ag nanorods with thin but dense and amorphous Al_2_O_3_ layers, and found that an Al_2_O_3_ layer of ~1.5 nm thick was effective to preserve the morphology of Ag nanorods to a temperature of 400 °C, and stabilize their SERS sensitivity in air for at least 50 days. The present study provides a simple way to fabricate Ag nanostructures with both excellent SERS sensitivity and outstanding SERS stability in air, which could be used in real SERS-based sensors.

## Methods

### Fabrication of Ag nanorod substrates

Slanted Ag nanorods were prepared on Si (001) substrates by oblique angle deposition (OAD) technique in an electron-beam system (GLAD, Thermionics Inc.) with a background vacuum level on the order of 10^–6^ Pa. During deposition, the incident angle of the vapor flux was set at ~86° off the surface normal of substrates, with a deposition rate of 0.75 nm/s. The deposition stopped at a thickness of 500 nm read by a quartz crystal microbalance (QCM). The detailed deposition procedure can be found elsewhere^49^,^50^,^51^,^52^.

### Atomic Layer Deposition of Al_2_O_3_ layers

Al_2_O_3_ layers were coated on as-prepared Ag nanorod in an ALD reactor (MNT-100, Wuxi MNT Micro and Nanotech Co.) at 50 °C. The Al_2_O_3_ precursors, i.e., trimethylaluminum (TMA, maintained at 150 °C) and water (maintained at 40 °C), were alternatively pumped into the reaction chamber using high purity N_2_ (99.999%, 15 sccm) as the carrier and purge gas. Typically, one complete reaction cycle took ~30 s, and consisted of four steps: (1) TMA reactant exposure time, 20 ms; (2) N_2_ gas purging time, 10 s; (3) water vapor exposure time, 10 ms; and (4) N_2_ gas purging time, 20 s. The thickness of alumina was controlled by varying the number of reaction cycles.

### Thermal Annealing

The as-prepared, uncoated and Al_2_O_3_-coated Ag nanorods were annealed in a quartz tube furnace at 200, 300 and 400 °C for 30 minutes in air.

### Characterization of Al_2_O_3_-coated Ag nanorods

The morphology, structure and chemical states of Ag nanorods and the Al_2_O_3_ layers were characterized by scanning electron microscope (SEM, JEOL-JMS-7001F), high-resolution transmission electron microscope (HRTEM, JEOL-2011) and X-ray photoelectron spectroscopy (XPS, PHI 5300) with Mg Kα as the excitation source, respectively. The reflectance spectra were measured by a R1 angle-resolved spectroscopy system (Idea Optics Co.) in the 200–1000 nm range, using a mirror-like Al film as the reference.

### SERS detections

The SERS measurements were conducted with an optical fiber micro-Raman system (i-Raman Plus, B&W TEK Inc.), using methylene blue (MB) and pyridine as the probing molecules. Before SERS measurements, the substrates (uncoated and coated Ag nanorods, before/after annealing) were merged into 5 × 10^−6^ M MB or 1 × 10^−2^ M pyridine aqueous solutions for 30 minutes, and dried naturally in air. The Raman spectra were obtained using a 785 nm laser as the excitation source, with its beam spot focused to ~80 μm in diameter and using an excitation power of 120 mW. The data collection time of one spectrum was set to 10 s. For every sample, the spectrum was obtained by averaging the spectra obtained from five different areas of the SERS substrate.

## Additional Information

**How to cite this article**: Ma, L. *et al.* Silver Nanorods Wrapped with Ultrathin Al_2_O_3_ Layers Exhibiting Excellent SERS Sensitivity and Outstanding SERS Stability. *Sci. Rep.* **5**, 12890; doi: 10.1038/srep12890 (2015).

## Supplementary Material

Supplementary Information

## References

[b1] Fleischmann M., Hendra P. J. & McQuillan A. J. Raman spectra of pyridine adsorbed at a silver electrode. Chem. Phys. Lett. 26, 163–166 (1974).

[b2] Michaels A. M., Nirmal M. & Brus L. E. Surface Enhanced Raman Spectroscopy of Individual Rhodamine 6G Molecules on Large Ag Nanocrystals. J. Am. Chem. Soc. 121, 9932–9939 (1999).

[b3] Nie S. & Emory S. R. Probing Single Molecules and Single Nanoparticles by Surface-Enhanced Raman Scattering. Science 275, 1102–1106 (1997). 9027306 10.1126/science.275.5303.1102

[b4] Zheng J. *et al.* Fabricating a Reversible and Regenerable Raman-Active Substrate with a Biomolecule-Controlled DNA Nanomachine. J. Am. Chem. Soc. 134, 19957–19960 (2012). 23190376 10.1021/ja308875r PMC3568521

[b5] Kneipp K. *et al.* Single Molecule Detection Using Surface-Enhanced Raman Scattering (SERS). Phys. Rev. Lett. 78, 1667 (1997).

[b6] Tabakman S. M., Chen Z., Casalongue H. S., Wang H. & Dai H. A New Approach to Solution-Phase Gold Seeding for SERS Substrates. Small 7, 499–505 (2011). 21360809 10.1002/smll.201001836 PMC3155739

[b7] Tawfick S. *et al.* Engineering of Micro- and Nanostructured Surfaces with Anisotropic Geometries and Properties. Adv. Mater. 24, 1628–1674 (2012). 22396318 10.1002/adma.201103796

[b8] Moskovits M. Surface-enhanced spectroscopy. Rev. Mod. Phys. 57, 783 (1985).

[b9] Kerker M. Electromagnetic model for surface-enhanced Raman scattering (SERS) on metal colloids. Accounts Chem. Res. 17, 271–277 (1984).

[b10] Schatz G. C. Theoretical studies of surface enhanced Raman scattering. Accounts Chem. Res. 17, 370–376 (1984).

[b11] Lombardi J. R., Birke R. L., Lu T. & Xu J. Charge-transfer theory of surface enhanced Raman spectroscopy: Herzberg-Teller contributions. J. Chem. Phys. 84, 4174–4180 (1986).

[b12] Bantz K. C. *et al.* Recent progress in SERS biosensing. Phys. Chem. Chem. Phys. 13, 11551–11567 (2011). 21509385 10.1039/c0cp01841d PMC3156086

[b13] Zhou Q., Li Z., Yang Y. & Zhang Z. Arrays of aligned, single crystalline silver nanorods for trace amount detection. J. Phys. D: Appl. Phys. 41, 152007 (2008).

[b14] Shanmukh S. *et al.* Rapid and sensitive detection of respiratory virus molecular signatures using a silver nanorod array SERS substrate. Nano Lett. 6, 2630–2636 (2006). 17090104 10.1021/nl061666f

[b15] Liz-Marzán L. M. Tailoring surface plasmons through the morphology and assembly of metal nanoparticles. Langmuir 22, 32–41 (2006). 16378396 10.1021/la0513353

[b16] Fernanda Cardinal M., Rodríguez-González B., Alvarez-Puebla R. A., Pérez-Juste J. & Liz-Marzán L. M. Modulation of localized surface plasmons and SERS response in gold dumbbells through silver coating. J. Phys. Chem. C 114, 10417–10423 (2010).

[b17] Jiang R., Chen H., Shao L., Li Q. & Wang J. Unraveling the evolution and nature of the plasmons in (Au core)-(Ag shell) nanorods. Adv. Mater. 24, P200–P207 (2012). 10.1002/adma.201201896 22714684

[b18] Pan C., Zhang Z., Su X., Zhao Y. & Liu J. Characterization of Fe nanorods grown directly from submicron-sized iron grains by thermal evaporation. Phys. Rev. B 70, 233404 (2004).

[b19] Alarifi H., Hu A., Yavuz M. & Zhou Y. N. Silver nanoparticle paste for low-temperature bonding of copper. J. Eelectron. Mater. 40, 1394 (2011).

[b20] Nanda K. K., Maisels A., Kruis F. E., Fissan H. & Stappert S. Higher surface energy of free nanoparticles. Phys. Rev. Lett. 91, 106102 (2003). 14525494 10.1103/PhysRevLett.91.106102

[b21] Jiang Q., Zhang S. H. & Li J. C. Grain size-dependent diffusion activation energy in nanomaterials. Solid State Commun. 130, 581 (2004).

[b22] Fang Z. Z. & Wang H. Densification and grain growth during sintering of nanosized particles. Int. Mater. Rev. 53, 326 (2008).

[b23] Formo E. V., Mahurin S. M. & Dai S. Robust SERS substrates generated by coupling a bottom-up approach and atomic layer deposition. ACS Appl. Mater. Inter. 2, 1987–1991 (2010).

[b24] Formo E. V., Wu Z., Mahurin S. M. & Dai S. *In Situ* High Temperature Surface Enhanced Raman Spectroscopy for the Study of Interface Phenomena: Probing a Solid Acid on Alumina. J. Phys. Chem. C 115, 9068–9073 (2011).

[b25] Whitney A. V., Elam J. W., Stair P. C. & Van Duyne R. P. Toward a thermally robust operando surface-enhanced Raman spectroscopy substrate. J. Phys. Chem. C 111, 16827–16832 (2007).

[b26] Liu M. *et al.* Is it possible to enhance Raman scattering of single-walled carbon nanotubes by metal particles during chemical vapor deposition? Carbon 80, 311 (2014).

[b27] Li X. *et al.* High-temperature surface enhanced Raman spectroscopy for *in situ* study of solid oxide fuel cell materials. Energ. Environ. Sci. 7, 306 (2014).

[b28] Bao L., Mahurin S. M. & Dai S. Controlled layer-by-layer formation of ultrathin TiO_2_ on silver island films via a surface sol-gel method for surface-enhanced Raman scattering measurement. Anal. Chem. 76, 4531–4536 (2004). 15283598 10.1021/ac049668c

[b29] John J. F., Mahurin S., Dai S. & Sepaniak M. J. Use of atomic layer deposition to improve the stability of silver substrates for *in situ*, high-temperature SERS measurements. J. Raman Spectrosc. 41, 4–11 (2010).

[b30] Im H., Lindquist N. C., Lesuffleur A. & Oh S. Atomic layer deposition of dielectric overlayers for enhancing the optical properties and chemical stability of plasmonic nanoholes. ACS Nano 4, 947–954 (2010). 20131870 10.1021/nn901842r

[b31] Bachenheimer L., Elliott P., Stagon S. & Huang H. Enhanced thermal stability of Ag nanorods through capping. Appl. Phys. Lett. 105, 213104 (2014).

[b32] Yang K., Liu Y., Hsu T. & Juang M. Strategy to improve stability of surface-enhanced raman scattering-active Ag substrates. J. Mater. Chem. 20, 7530–7535 (2010).

[b33] Zhang X., Zhao J., Whitney A. V., Elam J. W. & Van Duyne R. P. Ultrastable substrates for surface-enhanced Raman spectroscopy: Al_2_O_3_ overlayers fabricated by atomic layer deposition yield improved anthrax biomarker detection. J. Am. Chem. Soc. 128, 10304–10309 (2006). 16881662 10.1021/ja0638760

[b34] Mahurin S. M., Bao L. & Dai S. Controlled layer-by-layer formation of ultrathin oxide films on silver island films for surface-enhanced Raman scattering measurement. Isr. J. Chem. 46, 329–336 (2006). 10.1021/ac049668c 15283598

[b35] Xu H., Aizpurua J., Käll M. & Apell P. Electromagnetic contributions to single-molecule sensitivity in surface-enhanced Raman scattering. Phys. Rev. E 62, 4318 (2000). 10.1103/physreve.62.4318 11088961

[b36] Liu Y., Chu H. Y. & Zhao Y. Silver nanorod array substrates fabricated by oblique angle deposition: morphological, optical, and SERS characterizations. J. Phys. Chem. C 114, 8176–8183 (2010).

[b37] Whitney A. V. *et al.* Localized surface plasmon resonance nanosensor: a high-resolution distance-dependence study using atomic layer deposition. J. Phys. Chem. B 109, 20522–20528 (2005). 16853656 10.1021/jp0540656

[b38] Wilson C. A., Grubbs R. K. & George S. M. Nucleation and growth during Al_2_O_3_ atomic layer deposition on polymers. Chem. Mater. 17, 5625–5634 (2005).

[b39] Zhang L., Jiang H. C., Liu C., Dong J. W. & Chow P. Annealing of Al_2_O_3_ thin films prepared by atomic layer deposition. J. Phys. D: Appl. Phys. 40, 3707 (2007).

[b40] Xiong Y. *et al.* Electron cyclotron resonance plasma-assisted atomic layer deposition of amorphous Al_2_O_3_ thin films. Plasma Sci. Technol. 15, 52–55 (2013).

[b41] Yu J., Xiong J., Cheng B. & Liu S. Fabrication and characterization of Ag-TiO_2_ multiphase nanocomposite thin films with enhanced photocatalytic activity. Appl. Catal. B: Environ. 60, 211–221 (2005).

[b42] Stathatos E., Lianos P., Falaras P. & Siokou A. Photocatalytically deposited silver nanoparticles on mesoporous TiO_2_ films. Langmuir 16, 2398–2400 (2000).

[b43] Sugawara Y. *et al.* Localized and delocalized plasmons in metallic nanovoids. Phys. Rev. B 74, 245415 (2006).

[b44] Lesuffleur A., Im H., Lindquist N. C. & Oh S. Periodic nanohole arrays with shape-enhanced plasmon resonance as real-time biosensors. Appl. Phys. Lett. 90, 243110 (2007).

[b45] Qian L., Shen W., Shen B., Qin G. W. & Das B. Nanoporous gold-alumina core-shell films with tunable optical properties. Nanotechnology 21, 305705 (2010). 20603536 10.1088/0957-4484/21/30/305705

[b46] Lin X. D. *et al.* Synthesis of ultrathin and compact Au@ MnO_2_ nanoparticles for shell-isolated nanoparticle-enhanced Raman spectroscopy (SHINERS). J. Raman Spectrosc. 43, 40–45 (2012).

[b47] Uzayisenga V. *et al.* Synthesis, Characterization, and 3D-FDTD Simulation of Ag@SiO_2_ Nanoparticles for Shell-Isolated Nanoparticle-Enhanced Raman Spectroscopy. Langmuir 28, 9140–9146 (2012). 22506587 10.1021/la3005536

[b48] Li J. *et al.* Synthesis and characterization of gold nanoparticles coated with ultrathin and chemically inert dielectric shells for SHINERS applications. Appl. Spectrosc. 65, 620–626 (2011). 21639983 10.1366/10-06140

[b49] Zhang X., Zhou Q., Huang Y., Li Z. & Zhang Z. The regulation of surface-enhanced raman scattering sensitivity of silver nanorods by silicon sections. J. Nanomater. 2013, 72 (2013).

[b50] Zhang X., Zhou Q., Huang Y., Li Z. & Zhang Z. The nanofabrication and application of substrates for surface-enhanced Raman scattering. Int. J. Spectro. 2012 (2012).

[b51] Zhou Q., Liu Y., He Y., Zhang Z. & Zhao Y. The effect of underlayer thin films on the surface-enhanced Raman scattering response of Ag nanorod substrates. Appl. Phys. Lett. 97, 121902 (2010).

[b52] Zhou Q., Yang Y., Ni J., Li Z. & Zhang Z. Rapid recognition of isomers of monochlorobiphenyls at trace levels by surface-enhanced Raman scattering using Ag nanorods as a substrate. Nano Res. 3, 423–428 (2010).

