# Supplementary material for: Silver Nanorods Wrapped with Ultrathin Al2O3 Layers Exhibiting Excellent SERS Sensitivity and Outstanding SERS Stability
Source: Sci Rep. 2015 Aug 12;5:12890. doi: 10.1038/srep12890 (PMC4533008; doi:10.1038/srep12890)
Supplement: Supplementary Information [file srep12890-s1.pdf]

Silver Nanorods Wrapped with Ultrathin Al_2O_3 Layers Exhibiting Excellent SERS Sensitivity and Outstanding SERS Stability

Lingwei Ma[1], Yu Huang[1], Mengjing Hou[1], Zheng Xie[1, 3], & Zhengjun Zhang[2]*

[1]State Key Laboratory of New Ceramics and Fine Processing, School of Materials Science and Engineering, Tsinghua University, Beijing 100084, P.R. China

[2]Key Laboratory of Advanced Materials (MOE), School of Materials Science and Engineering, Tsinghua University, Beijing 100084, P.R. China

[3]High-Tech Institute of Xi'an, Shannxi 710025, P.R. China.

* Author to whom all correspondence should be addressed.

* E-mail: zjzhang@tsinghua.edu.cn

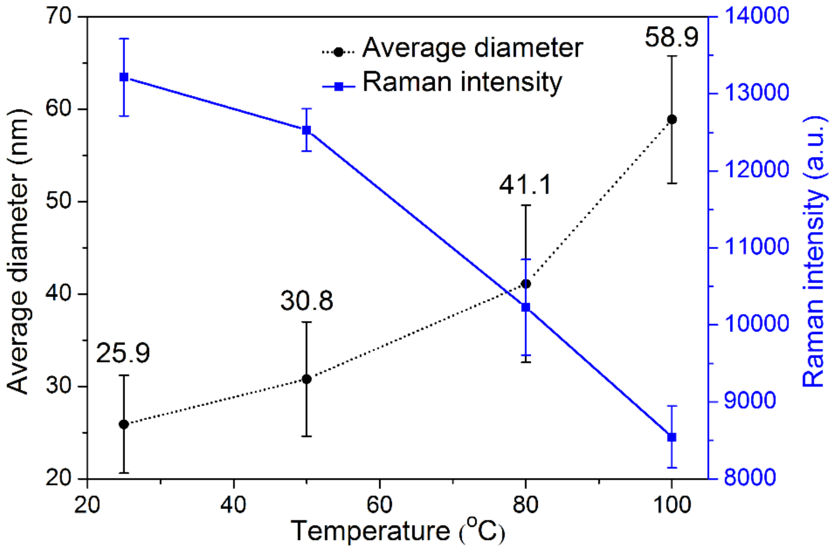

Figure S1. The average diameters and MB Raman intensities at 1622 cm^{-1} peak on Ag nanorods as a function of heating temperatures in the ALD chamber.

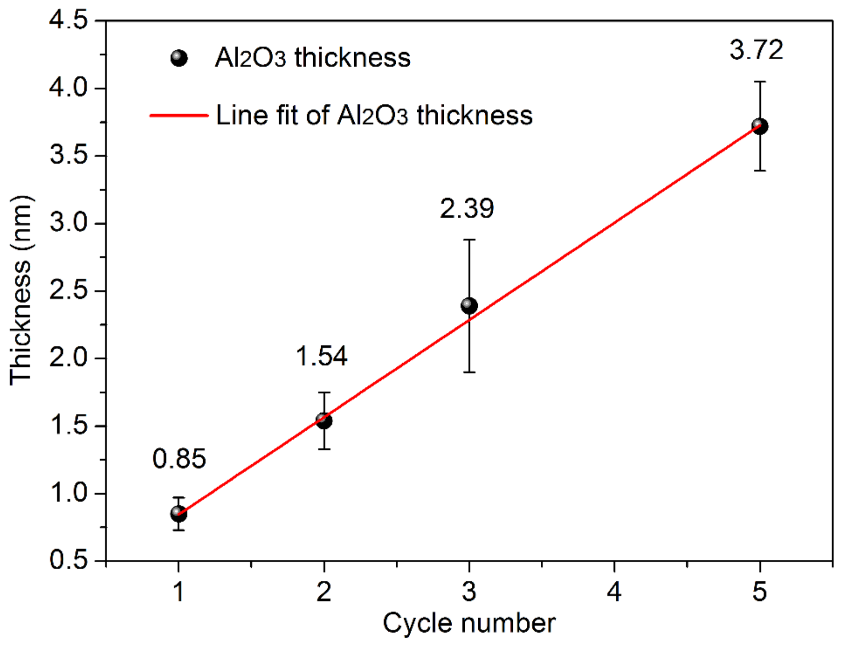

Figure S2. The plots of average thickness of Al_2O_3 layers as a function of ALD cycle numbers and the corresponding linear regression.

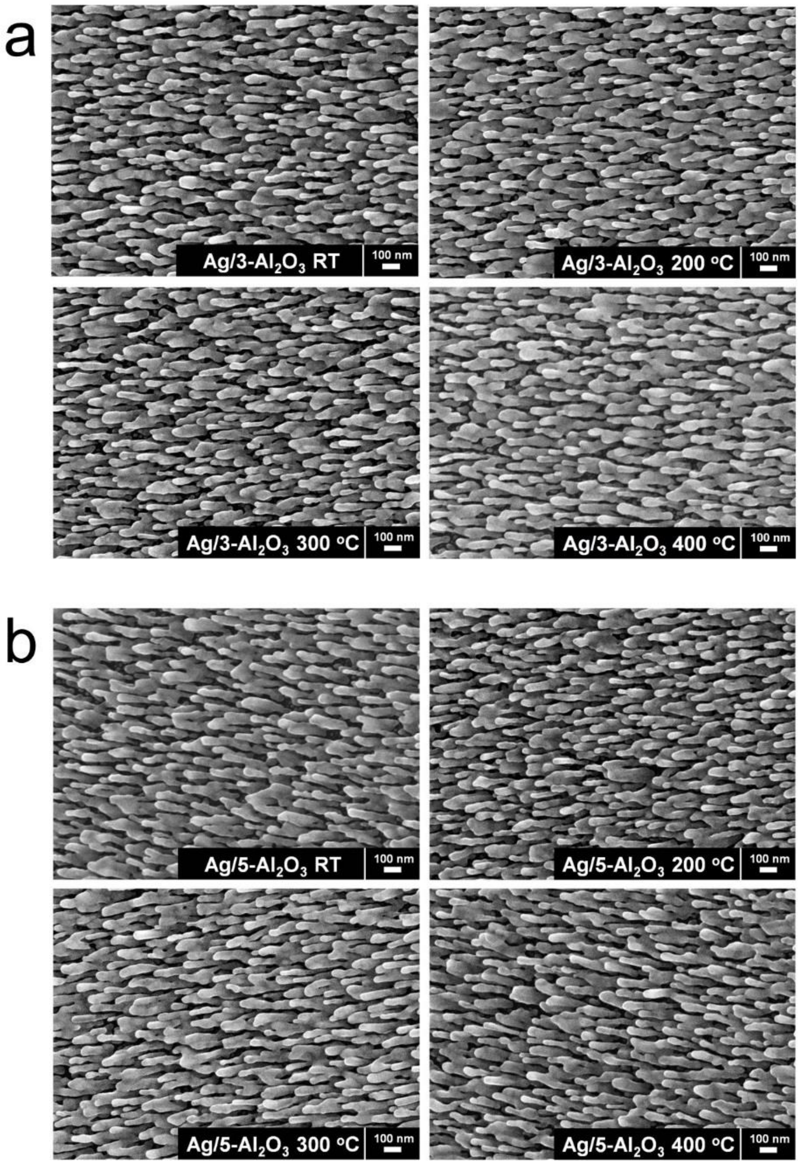

Figure S3. SEM images of Ag nanorods coated with Al_2O_3 layers by (a) 3 and (b) 5 ALD cycles before/after annealing at 200, 300 and 400 ℃, respectively.

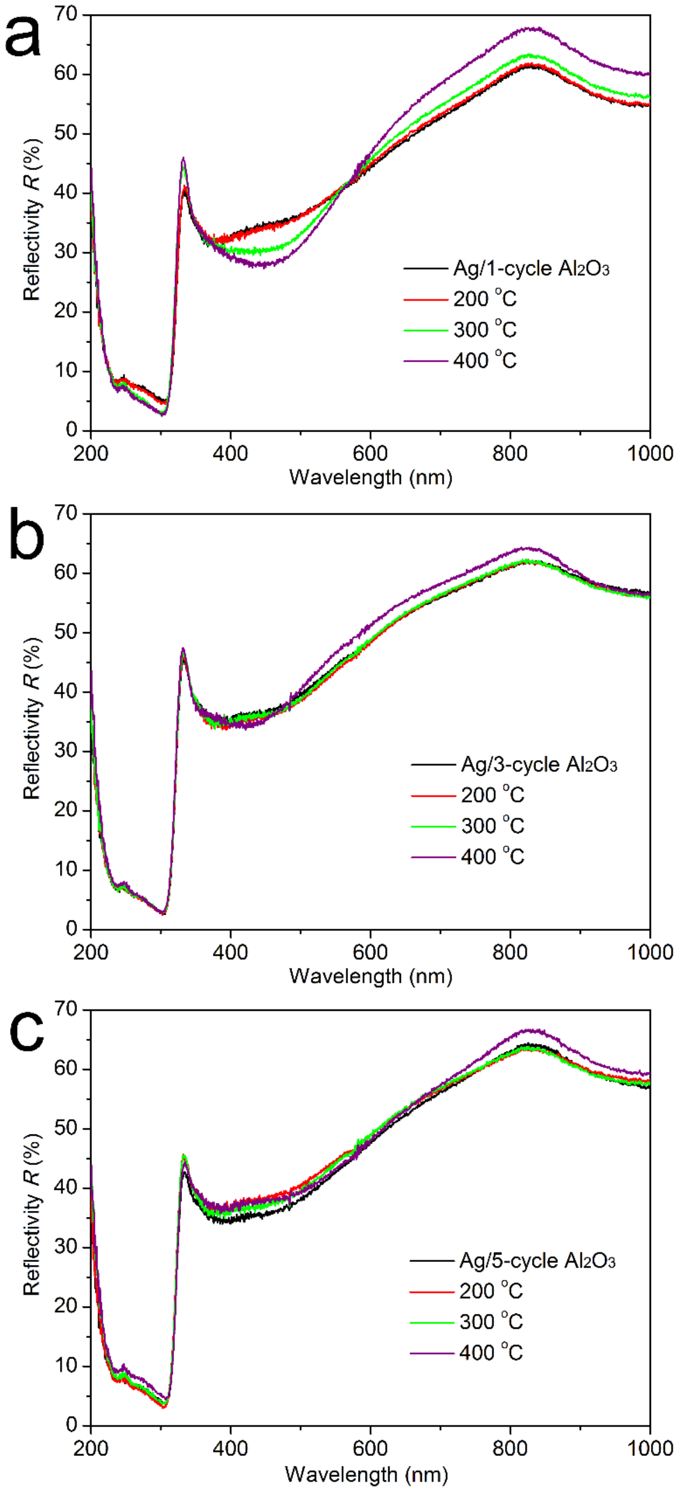

Figure S4. Reflectance spectra of Ag nanorods coated with Al_2O_3 layers by (a) 1, (b) 3 and (c) 5 ALD cycles before/after annealing at 200, 300 and 400 ℃, respectively.
